# Cyclodextrin-Catalyzed Organic Synthesis: Reactions, Mechanisms, and Applications

**DOI:** 10.3390/molecules22091475

**Published:** 2017-09-07

**Authors:** Chang Cai Bai, Bing Ren Tian, Tian Zhao, Qing Huang, Zhi Zhong Wang

**Affiliations:** 1 School of Pharmacy, Ningxia Medical University, Yinchuan 750004, China; changcaibai@163.com (C.C.B.); tianbingren1@163.com (B.R.T.); ZhaoTian7283@163.com (T.Z.); 2 Key Laboratory of Hui Ethnic Medicine Modernization, Ministry of Education, Yinchuan 750004, China

**Keywords:** cyclodextrin, catalysis, mechanism, green chemistry, organic reaction, asymmetric reaction

## Abstract

Cyclodextrins are well-known macrocyclic oligosaccharides that consist of α-(1,4) linked glucose units and have been widely used as artificial enzymes, chiral separators, chemical sensors, and drug excipients, owing to their hydrophobic and chiral interiors. Due to their remarkable inclusion capabilities with small organic molecules, more recent interests focus on organic reactions catalyzed by cyclodextrins. This contribution outlines the current progress in cyclodextrin-catalyzed organic reactions. Particular emphases are given to the organic reaction mechanisms and their applications. In the end, the future directions of research in this field are proposed.

## 1. Introduction

Supramolecular chemistry is defined as the chemistry of the intermolecular bond, and focuses on the chemical systems made up of a discrete number of assembled molecular subunits or components. It aims at developing highly complex chemical systems from molecular components reversibly held together by non-covalent intermolecular forces [1,2]. In recent years, the applications of supramolecular chemistry are of intriguing interest. The 2016 Nobel Prize in Chemistry was awarded to three organic chemists (Jean-Pierre Sauvage, Sir J. Fraser Stoddart, and Ben L. Feringa) for the designing and synthesis of molecular machines based on supramolecular chemistry [3,4,5]. In addition, reactive and catalysis represent significant features of the functional properties of supramolecular systems. Host molecules may chelate guest molecules (with definite stability, selectivity, and kinetic features), react with them (with definite rate, selectivity, turnover), and, finally, release the products, thus regenerating the reagents for a new cycle.

Cyclodextrins (CDs), as a model of the host molecule in supramolecular chemistry, are well-known macrocyclic oligosaccharides consisting of α-(1,4) linked glucose units [6,7]. They are inexpensive, water-soluble natural products, non-toxic, easily functionalized, and commercially available, and have been widely used as artificial enzymes, chiral separators, chemical sensors, and drug excipients [8,9,10,11,12,13,14], owing to their hydrophobic and chiral interiors [15,16,17]. Due to their remarkable inclusion capabilities with small organic molecules, more recent interests focus on organic reactions catalyzed by cyclodextrins. Parent cyclodextrins can be used as catalysts for organic synthesis, and can also be modified with some transition metals to form new catalysts, which catalyze certain organic reactions [18]. In addition, cyclodextrins and their derivatives are widely used as phase transfer catalysts for the synthesis of organic compounds [19]. Cyclodextrin-based supramolecular chemistry creates many research areas, and the present review focuses on the current progress in cyclodextrin-catalyzed organic reactions, mechanisms, and applications.

## 2. Application of Cyclodextrin in Conventional Reaction

### 2.1. Conventional Oxidation Reaction

**Scheme 1:**
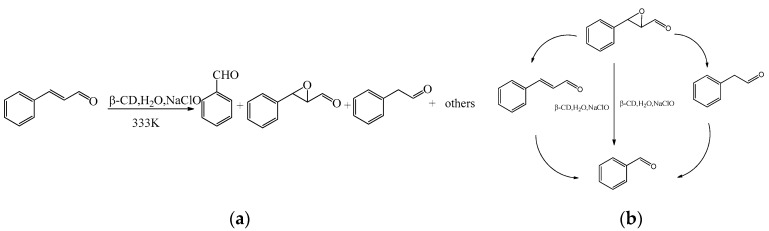
(**a**) The oxidation reaction of cinnamaldehyde catalyzed by β-CD; (**b**) the mechanism of oxidation of cinnamaldehyde catalyzed by β-CD.

The oxidation reaction is an important reaction in organic chemistry. In recent years, cyclodextrins have been widely used in the oxidation reaction. Cyclodextrins, as supramolecular catalysts, had the effect of an accelerated reaction rate so that the reaction time was decreased. Chen et al. [20] studied the oxidation reaction of cinnamicaldehyde using β-CD as the catalyst (Scheme 1a). Cinnamaldehyde and β-CD could form the inclusion complex with a molar ratio of 1:1 in water, and the rate of nucleophilic oxidation in the presence of β-CD was faster than that in the absence of the catalyst. In addition, the yield of benzaldehyde was up to 69% at 333 K. The proposed mechanism of the oxidation of cinnamicaldehyde was shown in Scheme 1b.

**Scheme 2:**
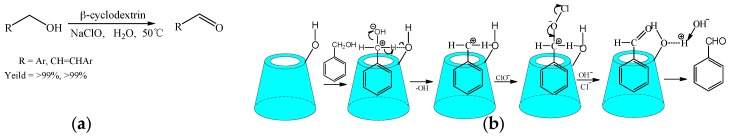
(**a**) The oxidation reaction of alcohol catalyzed by CDs; (**b**) the proposed reaction mechanism of alcohol oxidized to aldehyde.

Certainly, it is important and valuable that the reaction could be carried out under mild conditions, avoiding some side reactions. Ji et al. [21] studied the reaction of the alcohols oxidized to aldehydes under mild conditions, choosing the effective and inexpensive reactants (Scheme 2a). Because of the ability to form inclusion complexes between cyclodextrins and the reactants, the reaction solution was homogeneous, which was beneficial to the progress of the reaction. The substrate scope of the alcohols had been widely expanded to include aromatic alcohols in good yield. The advantages of these reactions consisted in inexpensive reactants and recycled catalysts of cyclodextrins. On the other hand, the mechanism of the oxidation of alcohols was proposed (Scheme 2b).

New cup-shaped α-cyclodextrin derivatives were designed and synthesized for the oxidation reaction. Lopez et al. [22] studied a series of new blocking-integrated or bridged cyclodextrins which had functional groups, such as 6^A^, 6^D^-di-*O*-(prop-2-methyl-1,3-dienyl), 6^A^, 6^D^-di-*O*-(prop-2-hydroxy-2-hydroxymethyl-1,3-dienyl)-hexadeca-*O*-benzyl, 6^A^, and 6^D^-di-*O*-(prop-2-formyl-2-hydroxy-1,3-dienyl)-hexadeca-*O*-benzyl. The results indicated that the derivatives could catalyze the oxidation reactions, i.e., 2-hydroxyaniline was oxidized into 2-amino-phenoxazin-2-one, catalyzed by the derivatives of cyclodextrins.

**Scheme 3:**
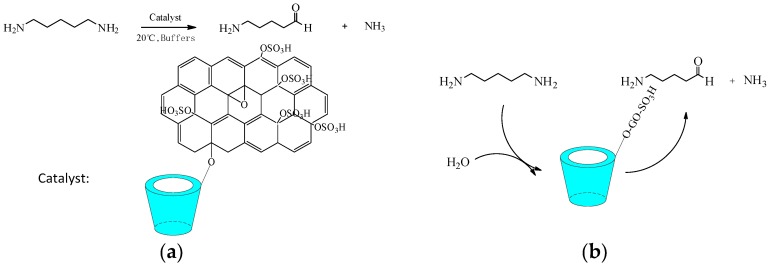
(**a**) The oxidation reaction of cadaverine catalyzed by β-CD/GO-SO_3_H–GCE; (**b**) the proposed mechanism of the oxidation of cadaverine.

Saghatforoush et al. [23] designed and prepared β-cyclodextrin-graphene oxide-SO_3_H composite-modified glassy carbon electrode (β-CD/GO-SO_3_H–GCE) as an electrochemical sensor, which could catalyze the oxidation reaction of cadaverine to 5-aminopentanal (Scheme 3a). Because the derivative of β-CD could accommodate cadaverine and promote its electron-transfer, the complexes provided a higher electroactive surface area. The main advantage of using the β-CD/GO-SO_3_H–GCE was easy and quick surface renewal after each use. On the other hand, the electrochemical sensor was successfully used to determine cadaverine in fish samples, and the electrode exhibited good repeatability and stability. Those results might open up a new approach for the use of the β-CD/GO-SO_3_H/GCE in the biochemical and medical analysis fields. The mechanism of the oxidation of cadaverine was proposed (Scheme 3b).

**Scheme 4:**
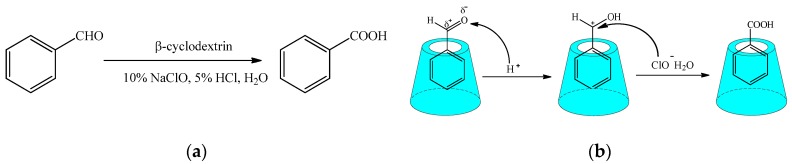
(**a**) The oxidation reaction of benzaldehyde catalyzed by β-CD; (**b**) the proposed mechanism of the oxidation reaction of benzaldehyde to benzoic acid.

In general, the aldehyde was directly oxidized to the carboxylic acid in the heterogeneous system. However, Shi et al. [24] studied the oxidation reaction with β-cyclodextrin in the homogeneous system. Benzaldehyde could be accommodated in the β-cyclodextrin cavity to form a complex, which was dissolved in the aqueous phase. Using the mild oxidizing agents (NaClO/HCl), benzaldehyde could be oxidized to benzoic acid, and the optimum reaction conditions were explored (Scheme 4a). Under the optimized condition, the yield was up to 98%. In this reaction, the reactants were inexpensive and β-cyclodextrin could be recycled. In addition, the corresponding mechanism was that the oxidizing agents oxidized benzaldehyde to carboxylic acid when the acid provided protons (Scheme 4b).

**Scheme 5:**
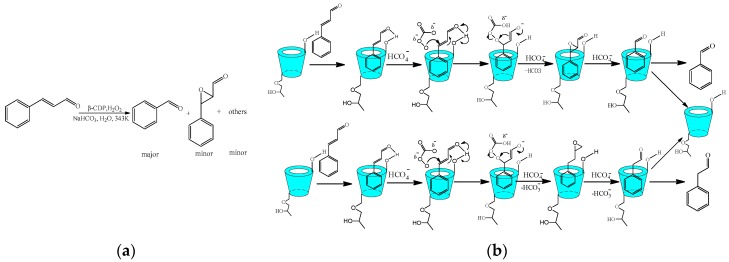
(**a**) The oxidation reaction of cinnamaldehyde to benzaldehyde catalyzed by β-CDP; (**b**) The proposed mechanism of the oxidation of cinnamaldehyde.

In the aspect of polymer of cyclodextrins, Yang et al. [25,26] designed and synthesized a new β-cyclodextrin cross-linked with chitosan (β-CDP) for the selective oxidation of cinnamaldehyde in the presence of bicarbonate and hydrogen peroxide (Scheme 5a). The experimental results clearly showed that β-CDP could form the complexes with cinnamaldehyde through the weak interaction of the hydrogen bond, and chitosan played an important role in the complexes. Under the optimal condition, benzaldehyde was obtained in 78% yield, compared to the parent cyclodextrin as the catalyst. In addition, the reusability of the catalyst was evaluated, and it could be recycled six times, which indicated that β-CDP was an effective catalyst for the selective oxidation of cinnamaldehyde. The advantages of the polymer of cyclodextrins consisted in simple preparation and convenient recycling, which made them useful for manufacturing natural benzaldehyde in an industrial process. The proposed mechanism of the oxidation of cinnamicaldehyde was shown in Scheme 5b, where β-CDP acted as PTC in the two-phase solution medium.

Similarly, Yang et al. [27] conjugated lignin to cyclodextrin to prepare a new material as a reverse phase transfer catalyst for the oxidation reaction of different alcohols to aldehydes in good yield. The catalyst was eco-friendly, and can be recycled. In addition, Chen et al. [28] found that the degrees of substitution of 2-HP-β-CD had an effect on the hydrolysis of cinnamaldehyde. When the degree of substitution increased, the rate of hydrolysis decreased.

### 2.2. Conventional Reduction Reaction

The processes of oxidation and reduction occur simultaneously, and the reduction reaction is also an important reaction in organic chemistry.

**Scheme 6:**
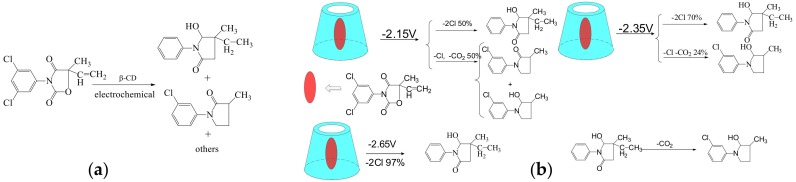
(**a**) The reduction reaction of 3-(3,5-dichlorophenyl)-5-methyl-5-vinyl-1,3-oxazolidine-2,4-dione catalyzed by β-CD; (**b**) the reduction mechanism of 3-(3,5-dichlorophenyl)-5-methyl-5-vinyl-1,3-oxazolidine-2,4-dione catalyzed by β-CD.

Under non-aqueous conditions, Pospisil et al. [29] studied the reduction reaction of 3-(3,5-dichlorophenyl)-5-methyl-5-vinyl-1,3-oxazolidine-2,4-dione using catalyzed by β-CD using the electrochemical method (Scheme 6a). The mechanism of the reduction of vinclozoline through the inclusion complex with β-CD was proposed (Scheme 6b).

**Scheme 7:**
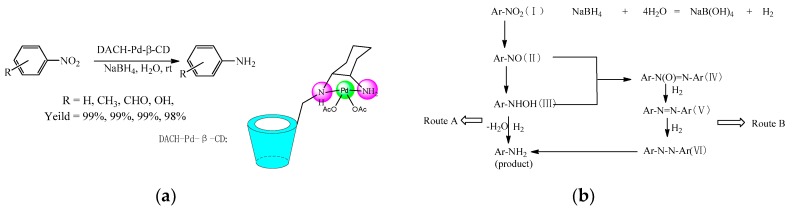
(**a**) The reduction reaction of nitroarene catalyzed by DACH-Pd-β-CD; (**b**) the proposed mechanism pathways of nitroarene reduction.

Guo et al. [30] modified cyclodextrin with palladium to synthesize a new material (DACH-Pd-β-CD) for the reduction reaction of a serial of nitrobenzene to aromatic amine compounds in excellent yield (Scheme 7a). On the other hand, the catalyst could be easily separated from the reaction solution, and still maintained high catalytic activity after five cycles. The mechanism of the reduction of nitrobenzene through the inclusion complex with DACH-Pd-β-CD was proposed (Scheme 7b).

**Scheme 8:**
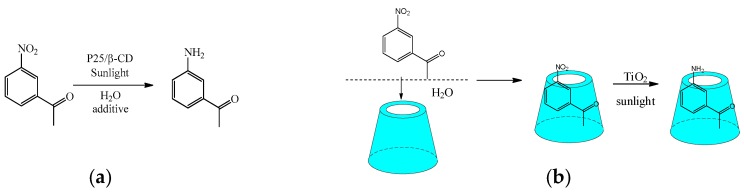
(**a**) The photoreduction of 3-nitroacetophenone catalyzed by β-CD-TiO_2_; (**b**) the mechanism of the photoreduction of 3-nitroacetophenone catalyzed by β-CD-TiO_2_.

Kakroudi et al. [31] constructed the host-guest system using β-cyclodextrin and nanosized-titania for the photocatalytic reduction of nitroaromatic compound under sunlight (Scheme 8a). This system, called “Green Nest”, was eco-friendly, and highly efficient to the reduction reaction. Also, more interestingly, nitroaromatic compounds could be reduced to aromatic amine compounds through one-pot reaction. The mechanism of the reduction of the nitroaromatic compound through the inclusion complex with β-cyclodextrin was proposed (Scheme 8b).

**Scheme 9:**
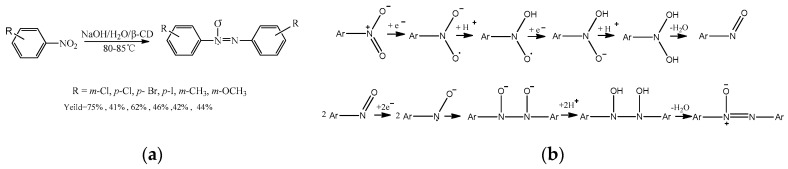
(**a**) The reduction reaction of nitrobenzene catalyzed by β-CD; (**b**) the proposed mechanism pathways of nitrobenzene reduction.

Lu et al. [32] studied the reduction reaction of nitrobenzene in the presence of sodium hydroxide and β-cyclodextrin (Scheme 9a). β-Cyclodextrin could form the inclusion complex with nitrobenzene. In the aqueous solution, the mechanism of the reduction of nitrobenzene through the one-electron transfer process was proposed (Scheme 9b).

### 2.3. Conventional Addition Reaction

An addition reaction, one of the important reactions in organic chemistry, makes two or more molecules combine to form a larger one.

**Scheme 10:**
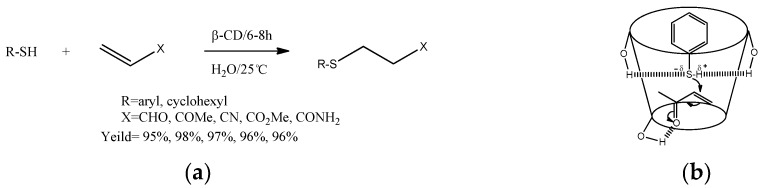
(**a**) The addition reaction of thiols with olefins catalyzed by β-CD; (**b**) the proposed mechanism of thiols with olefins catalyzed by β-CD.

Krishnaveni et al. [33,34] studied the addition reaction of thiols with olefins in the presence of β-cyclodextrin under room temperature (Scheme 10a), and the yield was higher (up to 98%) than that in the absence of β-cyclodextrin. In addition, the advantages of this method consisted in a lower reaction time and the recycling of the catalyst. The mechanism of the addition of thiols with olefins through the inclusion complex with β-cyclodextrin was proposed (Scheme 10b). Similarly, Srinivas et al. [35] studied the Michael addition reaction of benzeneselenol with olefins, and the yield could achieve up to 88% under the optimal condition. In those addition reactions mediated by β-CD, the process was economical and eco-friendly.

**Scheme 11:**
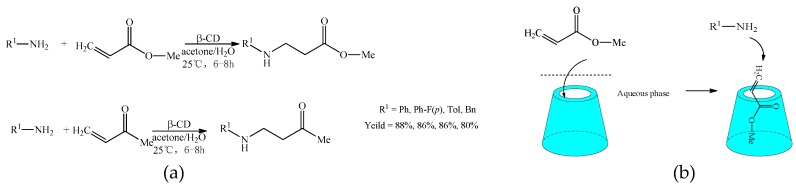
(**a**) The addition reaction of amines with alkenes catalyzed by β-CD; (**b**) the proposed mechanism of addition reaction of amines with alkenes.

In the aspect of the Aza-Michael addition reaction, Surendra et al. [36] studied the addition reaction of amines with alkenes in the presence of β-cyclodextrin (Scheme 11a). When the contents of β-cyclodextrin increased, the yields were improved. More interestingly, β-cyclodextrin (as the catalyst) could be recycled. The mechanism of the addition of amines with alkenes mediated by β-cyclodextrin was proposed (Scheme 11b).

**Scheme 12:**
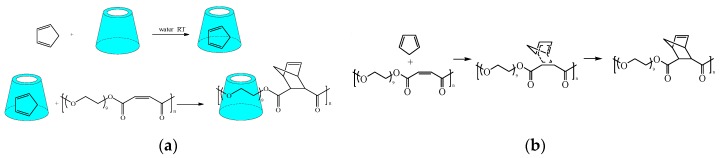
(**a**) The addition reaction of the unsaturated polyester catalyzed by β-CD; (**b**) the proposed mechanism of addition reaction of unsaturated polyester.

Regarding the Diels–Alder reaction, Alupei et al. [37] reported the addition reaction of cyclopentadiene with unsaturated polyester in the presence of methylated-β-cyclodextrin and synthesized a new type of polypseudorotaxane (Scheme 12a). The results indicated that the complex of cyclopentadiene with cyclodextrin was formed in water under room temperature, which greatly enhanced the stability and solubility of the monomer in water. Thus, the Diels–Alder reaction could be successfully carried out in an environmentally friendly manner by using the resulting complex of cyclopentadiene and methylated-β-cyclodextrin. The mechanism of the addition reaction of cyclopentadiene with unsaturated polyester through the inclusion complex with methylated-β-cyclodextrin was proposed (Scheme 12b).

**Scheme 13:**
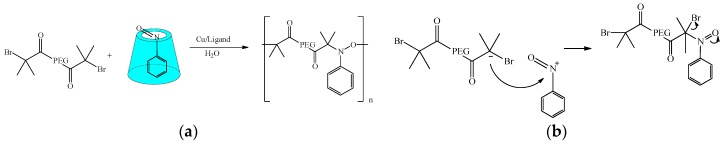
(**a**) The macromolecular addition reaction catalyzed β-CD; (**b**) the mechanism of the macromolecular addition reaction.

Condensation polymerization was a simple method for the synthesis of the sequence chain. Tao et al. [38] synthesized a hydrophilic periodic polymer using the nitroso benzene/cyclodextrin complex (Scheme 13a). Firstly, the water-soluble inclusion complexes of CD/NB were formed and then polymerized with poly (ethylene glycol) bis (α-bromoisobutyrate) to produce macromolecular polymers in good yield. The mechanism of this reaction was proposed (Scheme 13b). The process provided a simple method to synthesize the sequence chain and was beneficial to the application in industry.

**Scheme 14:**
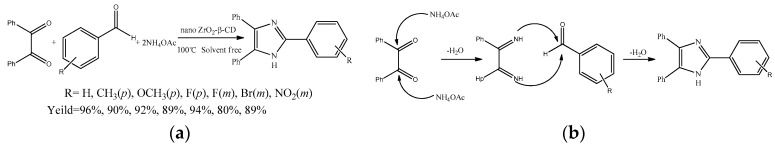
(**a**) The addition reaction of 2-phenyl imidazole derivatives catalyzed by ZrO_2_-β-cyclodextrin; (**b**) the mechanism of addition reaction of 2-phenyl imidazole derivatives.

Girish et al. [39] reported the synthesis of 2-phenyl imidazole derivatives by the addition reaction using nano ZrO_2_-β-cyclodextrin as a supramolecular catalyst in good yield (Scheme 14a). In addition, the catalyst can be recycled to facilitate resource conservation. The mechanism of the addition reaction of 2-phenyl imidazole derivatives was proposed (Scheme 14b).

**Scheme 15:**
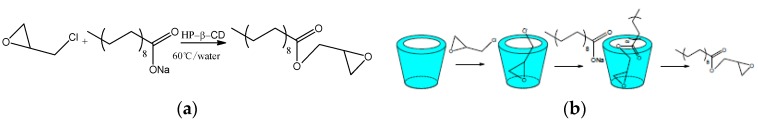
(**a**) The synthesis of glycidyl monostearate catalyzed by HP-β-CD; (**b**) the proposed mechanism of the synthesis of glycidyl monostearate.

Ge et al. [40] studied the synthesis of glycidyl monostearate using HP-β-CD as a biomimic catalyst (phase transfer catalyst) (Scheme 15a). HP-β-CD showed the highest catalytic activity, and the yield was up to 92.6% under the optimized reaction conditions. The kinetic profiles indicated that HP-β-CD acted as the catalyst by accommodating small molecules to form inclusion complexes (Scheme 15b).

Regarding polymerization, Galia et al. [41] reported the ring opening polymerization (ROP) of ε-caprolactone in the presence of β-cyclodextrin in batch reactors. It was interesting that the rate of the polymerization was enhanced, and the monomer conversion was up to 99% with wet β-CD under pressure. On the other hand, MALDI-TOF analyses indicated that a major fraction of polymer chains formed under pressure were initiated by water molecules. The experimental results indicated that the reaction mechanism in the presence of wet β-CD, similar to that for lipase-catalyzed ROP of cyclic esters, is operative, i.e., the secondary hydroxyl group of β-CD could mimic the role of the hydroxyl group of serine 105. The hydroxyl groups of β-CD brought a nucleophilic attack on the carbonyl of the cyclic ester resulting in the formation of a linear acyl complex, while the inner water in the cavity of β-CD acted as the role of water molecules bound to the hydration shell of the enzyme, resulting in the formation of an α, ω-hydroxy acid.

### 2.4. Conventional Substitution Reaction

Substitution reaction is a chemical reaction where one functional group in a molecule is replaced by another functional group.

**Scheme 16:**
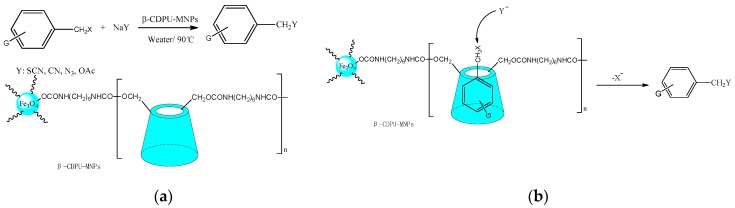
(**a**) The substitution reaction of benzyl halides catalyzed by β-CDPU-MNPs; (**b**) the proposed mechanism of the substitution reaction of benzyl halides.

Kiasat et al. [42] constructed the β-cyclodextrin-polyurethane, polymer-coated, Fe_3_O_4_ magnetic nanoparticle as a novel class of catalyst for the substitution reaction of benzyl halides in water (Scheme 16a). As a solid–liquid phase-transfer catalyst, it showed ability as a nucleophilic substitution reaction of benzyl halides with azide, cyanide, thiocyanate, and acetate anions. The possible mechanism of this reaction was proposed (Scheme 16b). It was more interesting that no evidence for the formation of by-products was observed and the targeted products were obtained in high purity without further purification. In addition, the nanomagnetic polymer brush catalyst was readily removed from the solution through the application of a magnetic field, thus allowing straightforward reuse.

**Scheme 17:**
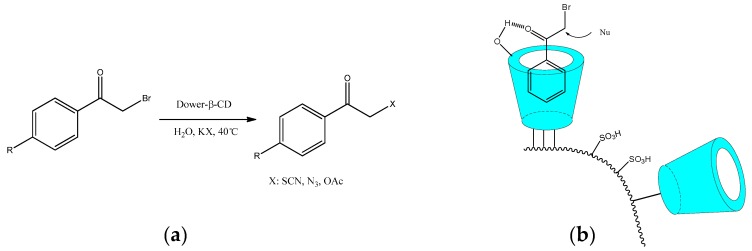
(**a**) The substitution reaction of alkyl halides catalyzed by Dower-β-CD; (**b**) the proposed mechanism of the substitution reaction of alkyl halides.

Kiasat et al. [43] developed the substitution reaction of phenacyl derivatives in water using β-cyclodextrin immobilized on Dowex resin as an efficient solid-liquid phase transfer catalyst (Scheme 17a). The nucleophilic substitution reactions were carried out under a mild reaction condition and given the products in good yields (80–95%). Also, the plausible reaction mechanism of alkyl halides catalyzed by Dower-β-CD was proposed (Scheme 17b). Furthermore, the catalyst could be recycled and reused by convenient separation with little loss of activity.

### 2.5. Conventional Hydrolysis Reaction

**Scheme 18:**
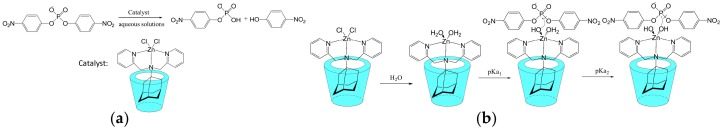
(**a**) The hydrolysis reaction of bis(4-nitrophenyl)phosphate catalyzed by β-cyclodextrin; (**b**) the mechanism of the hydrolysis reaction of bis(4-nitrophenyl)phosphate.

Cyclodextrins are widely applied to the hydrolysis reaction in organic chemistry. Zhou et al. [44] constructed the zinc (II) inclusion complexes using β-CD as a host molecule, in which the guest molecule with the adamantane pendant was inserted into the cavity of β-CD. As the model of metallohydrolase (Scheme 18a), the pH behavior of the rate constant of bis (4-nitrophenyl) phosphate hydrolysis exhibited an obvious increase with the second-order rate constant (2.68 × 10^−3^ M^−1^ s^−1^) assigned to the di-hydroxo species, which was almost an order of magnitude higher than that of the reported mono-Zn (II)-hydroxo species. The enhanced reactivity probably was ascribed to the hydroxyl-rich microenvironment provided by β-CD, which might have an effect on the stability of the labile zinc-hydroxo species or the catalytic transition state (Scheme 18b).

**Scheme 19:**
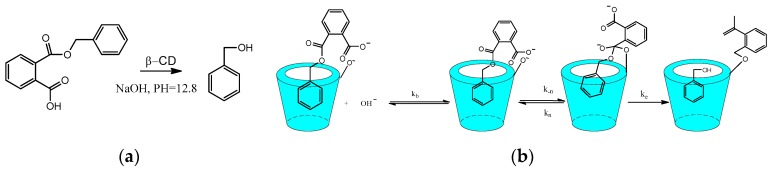
(**a**) The hydrolysis reaction of phthalate ester catalyzed by HP-β-CD; (**b**) the mechanism of the hydrolysis reaction of phthalate ester.

On the other hand, Andres et al. [45] studied the hydrolysis of phthalate ester in water in the presence of hydroxypropyl-β-cyclodextrin. The observed rate constant for the hydrolysis decreased in a non-linear fashion when the HP-β-CD concentration increased (Scheme 19a). The kinetic result was interpreted in terms of the formation of an inclusion complex of phthalate ester with HP-β-CD. In this complex, the carboxylate group formed hydrogen bonds with the hydroxyl groups at the rim of HP-β-CD, and the ionized group of HP-β-CD was far away from the reaction center due to electrostatic repulsion (Scheme 19b).

## 3. Application of Cyclodextrin in Asymmetric Reaction

### 3.1. Asymmetric Oxidation Reaction

Cyclodextrins have been used as chiral reaction containers for the asymmetric oxidation of aryl alkyl sulfides in moderate to poor enantiometric excesses by Drabowicz, Mikolajczyk [46], and Czarnik [47].

**Scheme 20:**
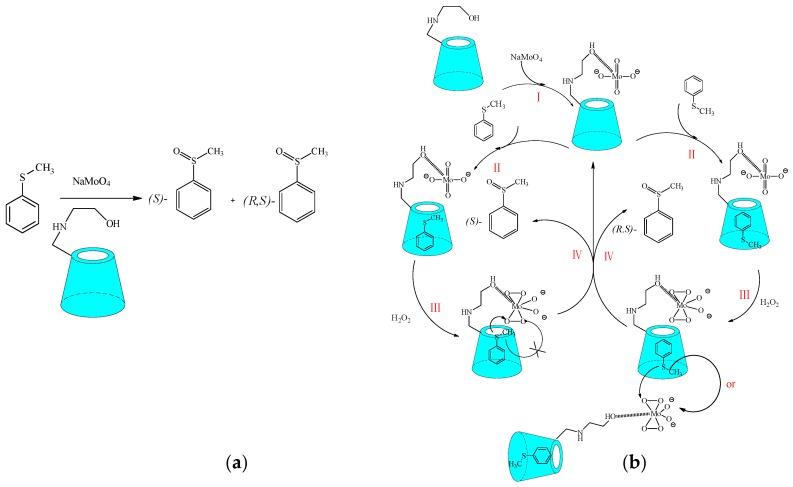
(**a**) The asymmetric oxidation of thioanisole catalyzed by modified β-cyclodextrin; (**b**) the proposed mechanism of the asymmetric oxidation of thioanisole.

In recent years, Shen et al. [48] designed and synthesized a serial of amino alcohol-modified β-cyclodextrins for asymmetric oxidation in water (Scheme 20a). Using the asymmetric oxidation of thioanisole as model reaction, the moderate ee value was achieved, which might be ascribed to the two different binding sites of modified β-cyclodextrin with thioanisole. The intramolecular catalysis produced (*S*)-methyl phenyl sulfoxide, while the intermolecular catalysis formed (*R*,*S*)-methyl phenyl sulfoxide. The delicate cooperation between the substituent groups and the parent β-CD played an important role in inducing enantioselectivity in the modified β-CDs (Scheme 20b). In addition, the enantioselectivity in the asymmetric oxidation of thioanisole catalyzed by modified β-CDs was illustrated through quantum calculation, which provided a practical method that explained the origin of the enantioselectivity in the asymmetric reaction.

**Scheme 21:**
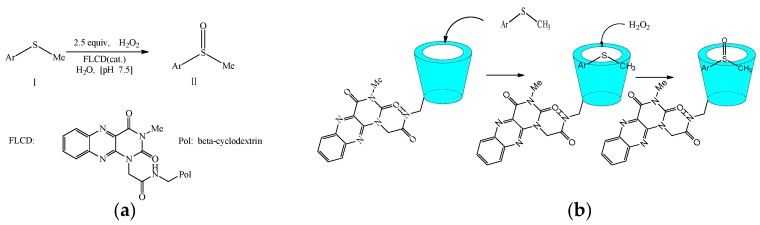
(**a**) The asymmetric oxidation of methyl phenyl sulfides catalyzed by β-cyclodextrin-flavin conjugates; (**b**) the proposed mechanism of the asymmetric oxidation of methyl phenyl sulfides.

Mojr et al. [49,50] synthesized two β-cyclodextrin-flavin conjugates as supramolecular catalysts for the sulfoxidation of methyl phenyl sulfides with H_2_O_2_ in water (Scheme 21a). The catalytic system, based on the β-cyclodextrin-alloxazine conjugate, had high enantioselectivity up to 80% ee. In addition, it was remarkable that the reaction was performed in water with very low loadings of the catalyst (low to 0.2 mol %) with a turnover number up to 395. The mechanism of the asymmetric oxidation of methyl phenyl sulfides through the inclusion complex with β-cyclodextrin-flavin conjugate was proposed (Scheme 21b).

### 3.2. Asymmetric Reduction Reaction

**Scheme 22:**
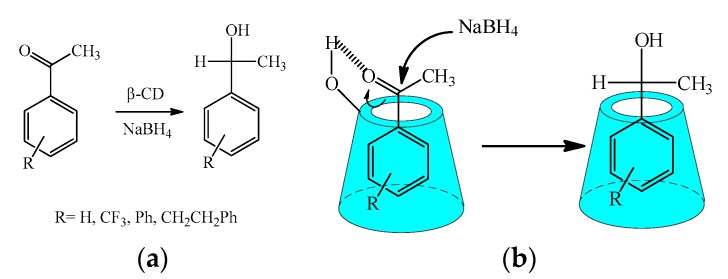
(**a**) The reduction reaction of ketones catalyzed by β-CD; (**b**) the proposed mechanism of the reduction reaction of ketones.

In the aspect of asymmetric reduction, Park et al. [51] studied the asymmetric reduction of various prochiral ketones with sodium borohydride using β-cyclodextrin and its derivatives as a chiral template (Scheme 22a) and found that the enantioselectivity in the asymmetric reduction of ketones to secondary alcohols was dependent on the structures of hosts and ketones, as well as the reaction temperature. In addition, the results indicated that the absolute configuration of the products depended on the conformational structure of the guest-host complexes, and the decrease of the degrees of freedom of the guest in the complexes was a major factor for the improvement of enantioselectivity (Scheme 22b).

**Scheme 23:**
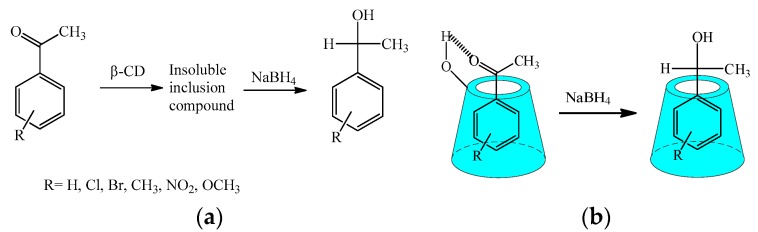
(**a**) The reduction reaction of substituted acetophenones catalyzed by β-CD; (**b**) the reduction mechanism of substituted acetophenones catalyzed by β-CD.

Deratani et al. [52] reported the effect of substituent groups on the asymmetric reduction induced by β-cyclodextrin in the reduction of a series of *o*-, *m*-, and *p*-substituted acetophenones (X = H, Br, C1, CH_3_, NO_2_, OCH_3_) with aqueous NaBH_4_ (Scheme 23a). The substitutions resulted in higher enantioselectivities than that obtained from acetophenone. On the other hand, acetophenone and its *m*- and *p*-derivatives produced mainly (−)-alcohol, while the *o*-derivatives gave the (+)-alcohol enantiomer. Enantioselectivity was mainly controlled by the shape and size of the β-cyclodextrin cavity in the case of the *o*-substituted acetophenones and governed by hydrophobic interactions in the case of the *m*-derivatives, while the asymmetic reduction of *p*-derivatives was complicated (Scheme 23b).

Similarly, the asymmetric reductions of prochiral ketones were also reported in different enantiometric excesses using modified cyclodextrins as supramolecular catalysts [53,54].

In the metal catalysts containing cyclodextrins, Jouffroy et al. [55] constructed a serial of phosphane-phosphite chelators comprising α-cyclodextrin, which were applied in Rh-catalysed asymmetric hydrogenation and hydroformylation. The results indicated that the highly mobile chelate complexes gave poor enantioselectivity, while the more rigid ones gave significant ee values up to 92%.

### 3.3. Asymmetric Addition Reaction

**Scheme 24:**
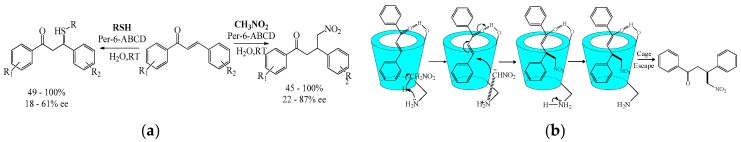
(**a**) The asymmetric Michael addition of nitromethane and thiols to chalcones catalyzed by Per-6-ABCD; (**b**) the proposed mechanism for the asymmetric Michael addition catalyzed by per-6-ABCD.

Cyclodextrin was also used in the asymmetric addition reaction. Pitchumani et al. [56] studied the asymmetric Michael addition of nitromethane and aliphatic thiols in aqueous media using per-6-amino-β-cyclodextrin (per-6-ABCD) as a chiral base catalyst (Scheme 24a). A better enantiomeric excess (60.6%) was observed in water at room temperature with good yield. The catalyst could be recovered and reused with little loss in its activity. The mechanism of the asymmetric addition of nitromethane and aliphatic thiols through the inclusion complex with per-6-ABCD was proposed (Scheme 24b). Using the same catalyst (per-6-ABCD), Pitchumani et al. [57] reported the asymmetric synthesis of 2-aryl-2,3-dihydro-4-quinolones with high yield (up to 99%) and enantiomeric excess (up to 99%).

**Scheme 25:**
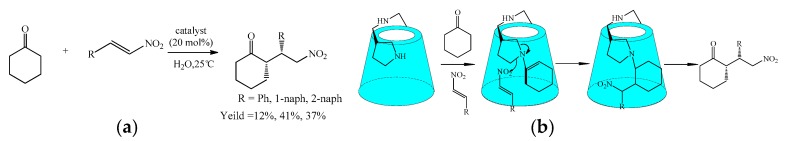
(**a**) The asymmetric Michael addition of cyclohexanone and 2-nitro-β-nitrostyrene catalyzed by (*S*)-2-aminomethylpyrrolidine-modified β-CD; (**b**) the proposed mechanism for the asymmetric Michael addition of cyclohexanone and 2-nitro-β-nitrostyrene.

Similarly, the asymmetric Michael addition of cyclohexanone and 2-nitro-β-nitrostyrene was reported by Zhu et al. [58]. Using (*S*)-2-aminomethylpyrrolidine-modified β-CD as the supramolecular catalyst (Scheme 25a), a good enantiomeric excess of 71% was observed in an aqueous buffer solution. The mechanism of the asymmetric Michael addition of cyclohexanone and 2-nitro-β-nitrostyrene was proposed (Scheme 25b).

**Scheme 26:**
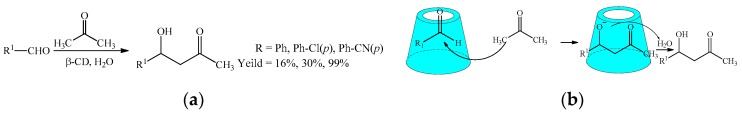
(**a**) The asymmetric aldol reaction of aldehydes and acetone catalyzed by β-CD; (**b**) the plausible mechanism of the asymmetric aldol reaction of aldehydes and acetone.

In the direct asymmetric aldol reactions of aldehydes and acetone using cyclodextrins as the supramolecular catalysts, Liu et al. [59] synthesized a new β-cyclodextrin-proline conjugated through a urea linker. Using the proline derived β-CD as the supramolecular catalyst (Scheme 26), a more excellent enantiomeric excess of 99% was observed in the moderate yield compared to that reported in the references [60,61]. In addition, the good recyclability and reusability of the catalyst was confirmed.

**Scheme 27:**
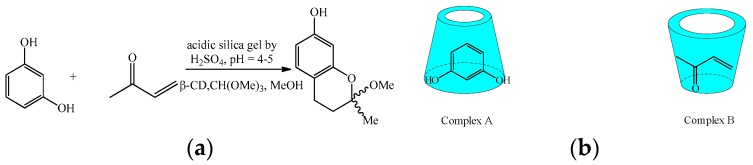
The asymmetric synthesis of (+)-2-methoxy-2-methylchroman-7-ol catalyzed by β-CD.

In the application of the asymmetric addition reaction based on cyclodextrins, Ni et al. [62] developed the asymmetric synthesis of (+)-2-methoxy-2-methylchroman-7-ol in solid-state using β-cyclodextrin as the chiral catalyst (Scheme 27). The chiral product was obtained from β-CD/1, 3-dihydroxylbenzene, and methyl vinyl ketone/β-CD in 82.8% yield and 78.4% ee.

## 4. Conclusions and Future Perspectives

Nowadays, the principles of supramolecular chemistry have broadly permeated through organic chemistry sub-disciplines besides organic synthetic methodology. The indiscernible, non-bonded, and bonding supramolecular interactions play a significant role in organic reactions and catalysis. Cyclodextrins belong to one of the most important and promising macrocyclic host molecules, since they are inexpensive, water-soluble, non-toxic, easily functionalized, and commercially available. In this mini-review, we focused on the cyclodextrin-based supramolecular systems for organic synthesis, and emphatically discussed the organic reaction mechanisms and their applications, including addition reactions, elimination reactions, substitution reactions, pericyclic reactions, rearrangement reactions, photochemical reactions, oxidation reactions, and reduction reactions. Among the above-mentioned organic reactions, the asymmetric synthesis was highlighted. The advantages of cyclodextrin-based supramolecular catalysts consisted in their facile preparation from inexpensive and easily available starting materials, their simple isolation, and their possibility of reuse. In addition, easy performance of the reaction under mild aqueous conditions brings great benefits to ecologically sustainable chemical processes and technologies. At present, considerable interest has been aroused in the functionization of cyclodextrins as enantioselective hosts for organic reactions, and cyclodextrin-based supramolecular chemistry will continually have an impact on organic synthetic methodology.

## References

[1] Lehn J.M. (1993). Supramolecular chemistry. Science.

[2] André J.M. (2014). The Nobel Prize in chemistry 2013. Chem. Int..

[3] The 2016 Nobel Prize in Chemistry. http://www.nobelprize.org/nobel_prizes/chemistry/laureates/2016/press.html.

[4] Leigh D.A. (2016). Genesis of the Nanomachines: The 2016 Nobel Prize in Chemistry. Angew. Chem. Int. Ed..

[5] Rhodes C.J. (2016). The 2016 Nobel Prize for Chemistry, Awarded For: “The Design and Synthesis of Molecular Machines”. Sci. Prog..

[6] De P.E., Cereda C.M., Fraceto L.F., de Araújo D.R., Franz-Montan M., Tofoli G.R., Ranali J., Volpato M.C., Groppo F.C. (2012). Micro and nanosystems for delivering local anesthetics. Expert Opin. Drug Deliv..

[7] Ain S., Kumar B., Pathak K. (2015). Cyclodextrin: Versatile carrier in drug formulations and delivery systems. Int. J. Pharm. Chem. Biol. S.

[8] Cui L., Liu Y., Liu T., Yahong Y., Tianli Y., Rui C., Zhouli W. (2017). Extraction of epigallocatechin gallate and epicatechin gallate from tea leaves using β-Cyclodextrin. J. Food Sci..

[9] Aytac Z., Ipek S., Durgun E., Tekinay T., Uyar T. (2017). Antibacterial electrospun zein nanofibrous web encapsulating thymol/cyclodextrin-inclusion complex for food packaging. Food Chem..

[10] Concheiro A., Alvarez-Lorenzo C. (2013). Chemically cross-linked and grafted cyclodextrin hydrogels: From nanostructures to drug-eluting medical devices. Adv. Drug Deliv. Rev..

[11] Ao M., Gan C., Shao W., Xing Z., Yong C. (2016). Effects of cyclodextrins on the structure of LDL and its susceptibility to copper-induced oxidation. Eur. J. Pharm. Sci..

[12] Rathi V.K., Kesselheim A.S., Ross J.S. (2016). The US Food and Drug Administration 515 Program Initiative: Addressing the evidence gap for widely used, high-risk cardiovascular devices?. JAMA Cardiol..

[13] Sun T., Wang Q., Bi Y., Chen X., Liu L., Ruan C., Zhao Z., Jiang C. (2017). Supramolecular amphiphiles based on cyclodextrin and hydrophobic drugs. J. Mater. Chem. B.

[14] Rutenberg R., Leitus G., Fallik E., Poverenov E. (2016). Discovery of a non-classic host guest complexation mode in a β-cyclodextrin/propionic acid model. Chem. Commun..

[15] Ikeda H., Sugiyama T., Ueno A. (2007). New chemosensor for larger guests based on modified cyclodextrin bearing seven hydrophobic chains each with a hydrophilic end group. J. Incl. Phenom. Macrocycl. Chem..

[16] Roy M.C., Roy M.N. (2015). Investigation of Inclusion Complex formed by Ionic Liquid and β-Cyclodextrin through Hydrophilic and Hydrophobic Interactions. RSC Adv..

[17] Cavalieri F., El H.A., Chiessi E., Paradossi G., Villa R., Zaffaroni N. (2006). Tethering functional ligands onto shell of ultrasound active polymeric microbubbles. Biomacromolecules.

[18] Hapiot F., Bricout H., Menuel S., Tilloy S., Monflier E. (2014). Recent breakthroughs in aqueous cyclodextrin-assisted supramolecular catalysis. Catal. Sci. Technol..

[19] Reetz M.T., Momotake A., Frömbgen C. (1999). Chemoselective Reduction of Halo-Nitro Aromatic Compounds by β-Cyclodextrin-Modified Transition Metal Catalysts in a Biphasic System. Synthesis.

[20] Chen H.Y., Ji H.B. (2011). β-cyclodextrin promoted oxidation of cinnamaldehyde to natural benzaldehyde in water. Chin. J. Chem. Eng..

[21] Ji H.B., Shi D.P., Shao M., Li Z., Wang L.F. (2005). Transition metal free and substrate-selective oxidation of alcohols using water as an only solvent in the presence of β-cyclodextrin. Tetrahedron Lett..

[22] Lopez O.L., Marinescu L., Bols M. (2007). New cup-shaped α-cyclodextrin derivatives and a study of their catalytic properties in oxidation reactions. Tetrahedron.

[23] Saghatforoush L., Hasanzadeh M., Shadjou N. (2014). β-cyclodextrin/graphene oxide grafted sulfonic acid: Application for electro-oxidation and determination of cadaverine in fish samples. J. Electroanal. Chem..

[24] Shi D.P., Ji H.B. (2009). β-cyclodextrin promoted oxidation of aldehydes to carboxylic acids in water. Chin. Chem. Lett..

[25] Yang Z., Zeng H., Zhou X., Ji H. (2013). Enhanced catalytic activity and recyclability for oxidation of cinnamaldehyde catalysed by β-cyclodextrin cross-linked with chitosan. Supramol. Chem..

[26] Yang Z., Zeng H., Zhou X., Ji H. (2012). Mechanism into selective oxidation of cinnamaldehyde using β-cyclodextrin polymer as phase-transfer catalyst. Tetrahedron.

[27] Yang Z., Zhang X., Yao X., Fang Y., Chen H., Ji H. (2016). β-cyclodextrin grafted on lignin as inverse phase transfer catalyst for the oxidation of benzyl alcohol in H_2_O. Tetrahedron.

[28] Chen H., Ji H. (2014). Effect of substitution degree of 2-hydroxypropyl-β-cyclodextrin on the alkaline hydrolysis of cinnamaldehyde to benzaldehyde. Supramol. Chem..

[29] PospíŠil L., Sokolová R., Hromadová M., Giannarelli S., Fuoco R., Colombini M.P. (2001). Inclusion complex of fungicide vinclozoline and β-cyclodextrin: The influence of host–guest interaction on the reduction mechanism. J. Electroanal. Chem..

[30] Guo Y., Li J., Zhao F., Lan G., Li L., Liu Y., Si Y., Jiang Y., Yang B., Yang R. (2016). Palladium-modified functionalized cyclodextrin as an efficient and recyclable catalyst for reduction of nitroarenes. RSC Adv..

[31] Kakroudi M.A., Kazemi F., Kaboudin B. (2014). β-cyclodextrin–TiO_2_: Green nest for reduction of nitroaromatic compounds. RSC Adv..

[32] Yun L.U., Liu J., Garry D., Liu D., Liu B.O. (2006). Reduction of mononitroarenes by hydroxide ion in water catalyzed by β-cyclodextrin: Enhanced reactivity of hydroxide ion. Tetrahedron Lett..

[33] Krishnaveni N.S., Surendra K., Rao K.R. (2005). Study of the Michael addition of β-cyclodextrin-thiol complexes to conjugated alkenes in water. Chem. Commun..

[34] Surendra K., Krishnaveni N.S., Reddy M.A., Nageswar Y.V.D., Rao K.R. (2003). Mild oxidation of alcohols with o-iodoxybenzoic acid (IBX) in water/acetone mixture in the presence of β-cyclodextrin. J. Org. Chem..

[35] Srinivas B., Kumar V.P., Sridhar R., Reddy V.P., Rao K.R. (2009). β-cyclodextrin-promoted addition of benzeneselenol to conjugated alCkenes in water. Helv. Chim. Acta.

[36] Surendra K., Krishnaveni N.S., Sridhar R., Rao K.R. (2006). β-cyclodextrin promoted Aza-Michael addition of amines to conjugated alkenes in water. Tetrahedron Lett..

[37] Vassilov A., Zlatkova M. (2005). Cyclodextrins in polymer modification: Diels-Alder addition of cyclopentadiene/methylated-β-cyclodextrin complex on unsaturated polyester and formation of a new type of polypseudorotaxane. Macromol. Rapid Commun..

[38] Tao F., Wang Q. (2015). Aqueous radical addition-coupling polymerization using nitroso benzene/cyclodextrin complex for synthesis of hydrophilic periodic polymer. RSC Adv..

[39] Girish Y.R., Kumar K.S.S., Thimmaiah K.N., Rangappa K.S., Shashikanth S. (2015). ZrO_2_-β-cyclodextrin catalyzed synthesis of 2,4,5-trisubstituted imidazoles and 1,2-disubstituted benzimidazoles under solvent free conditions and evaluation of their antibacterial study. RSC Adv..

[40] Ge T., Zou C., Zuo C. (2015). Monitoring the effects of hydroxypropyl-β-cyclodextrin as a biomimic catalyst (phase transfer catalyst) for glycidyl monostearate synthesis. Ind. Eng. Chem. Res..

[41] Galia A., Scialdone O., Spanò T., Grazia Valenti M., Grignard B., Lecomte P., Monflier E., Tilloy S., Rousseau C. (2016). Ring opening polymerization of ε-caprolactone in the presence of wet β-cyclodextrin: Effect of the operative pressure and of water molecules in the β-cyclodextrin cavity. RSC Adv..

[42] Kiasat A.R., Nazari S. (2012). Magnetic nanoparticles grafted with β-cyclodextrin–polyurethane polymer as a novel nanomagnetic polymer brush catalyst for nucleophilic substitution reactions of benzyl halides in water. J. Mol. Catal. A Chem..

[43] Kiasat A.R., Zarinderakht N., Sayyahi S. (2012). β-Cyclodextrin immobilized onto dowex resin: A unique microvessel and heterogeneous catalyst in nucleophilic substitution reactions. Chin. J. Chem..

[44] Zhou Y.H., Chen L.Q., Tao J., Shen J.L., Gong D.Y., Yun R.R., Cheng Y. (2016). Effective cleavage of phosphodiester promoted by the zinc(II) and copper(II) inclusion complexes of β-cyclodextrin. J. Inorg. Biochem..

[45] Andrés G.O., Rossi R.H.D., To D., Rúveda E., Rossi R.A. (2003). Mechanism of phthalate ester hydrolysis in water and in cyclodextrin mediated reactions. Arkivoc.

[46] Drabowicz J., Mikołajczyk M. (1984). Asymmetric Oxidation of Sulfides to Sulfoxides Catalyzed by β-Cyclodextrin. Phosphorous Sulfur Relat. Elem..

[47] Anthony W. (1984). Czarnik Cyclodextrin-mediated chiral sulfoxidations. J. Org. Chem..

[48] Shen H.M., Ji H.B. (2012). Amino alcohol-modified β-cyclodextrin inducing biomimetic asymmetric oxidation of thioanisole in water. Carbohydr. Res..

[49] Mojr V., Ínsky M.B., Cibulka R., Kraus T. (2011). Alloxazine- cyclodextrin conjugates for organocatalytic enantioselective sulfoxidations. Org. Biomol. Chem..

[50] Mojr V., Herzig V., Ínsky M.B., Cibulka R., Kraus T. (2010). Flavin-cyclodextrin conjugates as catalysts of enantioselective sulfoxidations with hydrogen peroxide in aqueous media. Chem. Commun..

[51] Park K.K., Sim W.J., Park J.W. (1997). Asymmetric induction by β-cyclodextrins in NaBH_4_ reduction of ketones. J. Incl. Phenom. Macrocycl. Chem..

[52] Deratani A., Renard E. (1994). Substituent effects in the enantioselective reduction of acetophenones with NaBH_4_ in the presence of β-cyclodextrin. Chirality.

[53] Wan Y., Wang X., Liu N. (2014). Interaction of β-cyclodextrin as catalyst with acetophenone in asymmetric reaction: A theoretical survey. J. Mol. Model..

[54] Nishimura T., Nakajima M., Maeda Y., Uemura S., Takekuma S.I., Takekuma H., Yoshida Z.I. (2004). γ-cyclodextrin-bicapped C60 -mediated asymmetric reduction of ketones with NaBH_4_. Bull. Chem. Soc. Jpn..

[55] Jouffroy M., Sémeril D., Armspach D., Matt D. (2013). Phosphane-Phosphite Chelators Built on a α-Cyclodextrin Scaffold: Application in Rh-Catalysed Asymmetric Hydrogenation and Hydroformylation. Eur. J. Org. Chem..

[56] Suresh P., Pitchumani K. (2008). Per-6-amino-β-cyclodextrin catalyzed asymmetric Michael addition of nitromethane and thiols to chalcones in water. Tetrahedron Asymmetr..

[57] Kanagaraj K., Pitchumani K. (2013). Per-6-amino-β-cyclodextrin as a Chiral Base Catalyst Promoting One-Pot Asymmetric Synthesis of 2-Aryl-2,3-dihydro-4-quinolones. J. Org. Chem..

[58] Zhu Q., Shen H., Yang Z., Ji H. (2016). Biomimetic asymmetric Michael addition reactions in water catalyzed by amino-containing β-cyclodextrin derivatives. Chin. J. Catal..

[59] Liu K., Zhang G. (2015). ChemInform Abstract: Direct Asymmetric Aldol reactions in aqueous media catalyzed by a β-cyclodextrin-proline conjugate with a urea linker. Tetrahedron Lett..

[60] Doyagüez E.G., Fernández-Mayoralas A. (2012). Proline–cyclodextrin conjugates: Synthesis and evaluation as catalysts for aldol reaction in water. Tetrahedron.

[61] Shen Z., Ma J., Liu Y., Jiao C., Li M., Zhang Y. (2005). β-cyclodextrin-immobilized (4*S*)-phenoxy-(*S*)-proline as a catalyst for direct asymmetric aldol reactions. Chirality.

[62] Ni T., Pan J.H., Wang S.Y., Fang X.U., Tao Y.W., She Z.G., Lin Y.C. (2008). Asymmetric synthesis of 2-methoxy-2-methylchroman-7-ol in solid-state β-cyclodextrin complexes. Chin. J. Chem..

